# Strategy for addressing research-site overlap in pragmatic clinical trials: lessons learned from the NIH-DOD-VA Pain Management Collaboratory (PMC)

**DOI:** 10.1186/s13063-020-04941-8

**Published:** 2020-12-11

**Authors:** Mary Geda, Steven Z. George, Diana J. Burgess, Dylan V. Scarton, William T. Roddy, Kirsha S. Gordon, Paul F. Pasquina, Cynthia A. Brandt, Robert D. Kerns, Peter Peduzzi, on behalf of the NIH-DoD-VA Pain Management Collaboratory

**Affiliations:** 1 The Pain Management Collaboratory Coordinating Center, Yale University, New Haven, CT USA; 2 Department of Internal Medicine, Yale School of Medicine, 300 George Street, New Haven, CT 06511 USA; 3 Duke Clinical Research Institute and Department of Orthopaedic Surgery, Duke University, Durham, NC USA; 4 HSR&D Durham VA Health Care System, Durham, NC USA; 5 Minneapolis Veterans Affairs Healthcare System, Minneapolis, MN USA; 6 Department of Medicine, University of Minnesota, Minneapolis, MN USA; 7 The Center for Rehabilitation Sciences Research, Uniformed Services University of the Health Sciences, Bethesda, MD USA; 8 Walter Reed National Military Medical Center, Bethesda, MD USA; 9 The Henry M. Jackson Foundation for the Advancement of Military Medicine, Bethesda, MD USA; 10 VA Connecticut Healthcare System, West Haven, CT USA; 11 Department of Emergency Medicine, Yale School of Medicine, New Haven, CT USA; 12 Department of Psychiatry, Yale School of Medicine, New Haven, CT USA; 13 Department of Biostatistics, and Yale Center for Analytical Sciences, Yale School of Public Health, New Haven, CT USA

**Keywords:** Pragmatic clinical trials, Pain management, Research-site overlap, Recruitment, VA health care, DoD health care, Collaboratory

## Abstract

**Background:**

The Pain Management Collaboratory (PMC) is a multi-site network of pragmatic clinical trials (PCTs) focused on nonpharmacological approaches to pain management, conducted in health care systems of the US Department of Defense (DoD) and Department of Veterans Affairs (VA) and co-funded by the National Institutes of Health (NIH). Concerns about potential research-site overlap prompted the PMC investigator community to consider strategies to avert this problem that could negatively affect recruitment and contaminate interventions and thus pose a threat to trial integrity.

**Methods:**

We developed a two-step strategy to identify and remediate research-site overlap by obtaining detailed recruitment plans across all PMC PCTs that addressed eligibility criteria, recruitment methods, trial settings, and timeframes. The first, information-gathering phase consisted of a 2-month period for data collection from PIs, stakeholders, and ClinicalTrials.gov. The second, remediation phase consisted of a series of moderated conference calls over a 1-month time period to develop plans to address overlap. Remediation efforts focused on exclusion criteria and recruitment strategies, and they involved collaboration with sponsors and stakeholder groups such as the Military Treatment Facility Engagement Committee (MTFEC). The MTFEC is comprised of collaborating DoD and university-affiliated PIs, clinicians, and educators devoted to facilitating successful pragmatic trials in DoD settings.

**Results:**

Of 61 recruitment sites for the 11 PMC PCTs, 17 (28%) overlapped. Four PCTs had five overlapping Military Treatment Facilities (MTFs), and eight PCTs had 12 overlapping VA Medical Centers (VAMCs). We developed three general strategies to avoid research-site overlap: (i) modify exclusion criteria, (ii) coordinate recruitment efforts, and/or (iii) replace or avoid any overlapping sites. Potential overlap from competing studies outside of the PMC was apparent at 26 sites, but we were not able to confirm them as true conflicts.

**Conclusion:**

Proactive strategies can be used to resolve the issue of overlapping research sites in the PMC. These strategies, combined with open and impartial mediation approaches that include researchers, sponsors, and stakeholders, provide lessons learned from this large and complex pragmatic research effort.

## Background

Pragmatic clinical trials (PCTs) evaluate the effectiveness of interventions in real-world scenarios involving a broad set of participants, rather than in the more tightly controlled conditions set by explanatory trials that aim to confirm a specific physiological or clinical hypothesis [1, 2]. Research participants in PCTs are similar to individuals who would receive the test intervention if it became usual care, and they are designed to answer questions faced by patients, physicians, and policy makers [3]. Pragmatic approaches have gained traction in recent times to solve urgent health care problems such as the severe burden of chronic pain as well as to test and deliver non-addictive pain therapies to counter the nation’s opioid epidemic [4, 5]. The Pain Management Collaboratory (PMC) is a multi-site network of trials and investigators uniformly focused on pain management and co-funded by the National Institutes of Health (NIH), the Department of Defense (DoD), and the Department of Veterans Affairs (VA). The PMC is conducting 11 large-scale PCTs (Table 1) of nonpharmacological approaches for the management of pain and related conditions in DoD and VA health care delivery organizations.

**Table 1:** Pragmatic trials in the Pain Management Collaboratory (PMC)

PMC trial number	PMC trial name
1	Cooperative Pain Education and Self-Management: Expanding Treatment for Real-world Access (Copes ExTRA)
2	Engaging Veterans Seeking Service-Connection Payments in Pain Treatment
3	Chiropractic Care for Veterans, A Pragmatic Randomized Trial Addressing Dose Effects for cLBP (VERDICT)
4	Improving Veteran Access to Integrated Management of Low Back Pain (AIM-Back)
5	SMART Stepped Care Management for Low Back Pain in the Military Health System
6	Whole Health Team vs. Primary Care Group Education to Promote Non-Pharmacological Strategies to Improve Pain, Functioning,and Quality of Life in Veterans (*w*HOPE Study)
7	The APPROACH Trial: Assessing Pain, Patient Reported Outcomes and Complementary and Integrative Health (A VA National Demonstration Project)
8	Learning to Apply Mindfulness to Pain (LAMP)
9	Targeting Chronic Pain in Primary Care Settings Using Internal Behavioral Health Consultants
10	Ultrasound-Guided Percutaneous Peripheral Nerve Stimulation: A Non-Pharmacologic Alternative for the Treatment of Postoperative Pain
11	Resolving the Burden of Low Back Pain in Military Service Members and Veterans: A Multi-Site Pragmatic Clinical Trial (RESOLVETrial)

*cLBP* chronic low back pain, *VA* Veterans Affairs

Generally speaking, clinical sites best suited for PCTs are those with practical experience conducting multiple studies with a large number of patients. However, it is difficult for researchers to draw accurate conclusions if study participants receive more than one intervention targeting the same health condition. Thus, careful evaluation of competing studies in multi-site PCTs is important to avoid research-site overlap as well as to ensure that investigators can achieve adequately powered sample sizes from which to draw valid conclusions. Research-site overlap is particularly problematic for investigations of the effectiveness of various nonpharmacological treatment modalities (such as behavioral therapies) for chronic conditions that share symptoms such as back pain because it can be difficult to ascertain that co-enrollment has occurred. Various factors contribute to the potential for research-site overlap, which poses a threat to trial integrity.

The potential for research-site overlap was an early concern of the PMC. The phased timing of this effort—two PMC funding phases (2 years for planning and 4 years for implementation) and staggered PCT start dates—offered an important opportunity to avert potential co-enrollment of participants in multiple, concurrent trials (including non-PMC pain studies). In concert with PMC sponsors, the PMC Coordinating Center (PMC3, which supports the design and implementation of the PMC PCTs according to previously specified project milestones) oversaw this task of detecting, and resolving, overlap problems prior to final study design and participant recruitment/enrollment. Herein, we present effective strategies unique to PCTs that we used to address the problem of research-site overlap.

## Methods

### Information-gathering phase

To identify potential overlap, after notification of funding, PMC PCT principal investigators (PIs) provided the PMC3 with proposed target sites, eligibility criteria, and recruitment strategies. The PMC3 also conducted a search of ClinicalTrials.gov to identify potentially competing studies outside the PMC being conducted at target recruitment sites and shared this information with the PMC PIs. Recognizing the importance of a proactive approach for managing such a large group effort—and given that site-level information was often unavailable, outdated, or incomplete—the PMC3 developed a two-step strategy and implemented a second, remediation phase to resolve issues surrounding site selection.

#### Identifying overlapping sites within the PMC

Because PMC PIs had used different nomenclature when referring to their recruitment sites, the PMC3 standardized each location using either the VA-designated Facility name and Station ID number or the Health System Name and Certification Number designated by the US Department of Health and Human Services (HHS) Centers for Medicare and Medicaid Services (CMS). This process identified 61 sites as targets for PMC trials, and this information was shared with PIs in an Excel-based pivot table that could be filtered by site location or PMC PI. Creating a searchable format was especially useful for those PIs who were still actively selecting sites.

#### Obtaining eligibility criteria and recruitment plans within the PMC

The PMC3 contacted PMC PIs to obtain detailed recruitment plans—including eligibility criteria, recruitment methods, trial settings, and timeframes—to further inform which PCTs might have overlap issues. The PMC3 observed that because studies could be recruiting from different clinical settings within the same health care system (e.g., outpatient vs. inpatient), these and other studies with distinct pain populations, unique recruitment settings, and/or differing timeframes would be unlikely to need any remediation plan.

#### Identifying competing pain studies external to the PMC

Because interventional pain trials external to the PMC could also affect recruitment efforts, the PMC3 conducted a search of ClinicalTrials.gov to identify ongoing trials involving pain interventions (including opioid-tapering studies) that could interfere with PMC trial recruitment of similar patients in the same settings. This search identified 49 non-PMC pain trials with the potential for overlap at 26 PMC sites. Information describing these trials was incorporated into the Excel spreadsheet and included site name, condition being studied (e.g., post-operative pain), type of intervention, trial start date, trial completion date, title of the study, and National Clinical Trial (NCT) number. Further curation of this information by PCT PIs ascertained the expected impact of overlap.

To ascertain real-world circumstances across recruitment sites, the PMC3 sought and obtained additional information from key stakeholders within PMC sponsoring agencies, to ensure that PMC interventions and data collection did not interfere with ongoing agency efforts. For example, VA representatives provided information about the rollout of its *Whole Health Evaluation Program*, which expands the availability of complementary and integrative health services [6] to meet Congress’ 2016 Comprehensive Addiction and Recovery Act mandate to provide nonpharmacological complementary and integrative health options for chronic pain. During the PMC PCT planning phase, the VA administered a national survey of 21,000 chronic pain patients from 2018 through 2019 at 18 flagship Veterans Administration Medical Centers (VAMCs), and PMC PIs agreed not to recruit from these institutions until data collection was complete.

A unique feature of the PMC is the formation and involvement of the Military Treatment Facility Engagement Committee (MTFEC) comprised of collaborating DoD and university-affiliated PIs, clinicians, and educators devoted to facilitating successful pragmatic trials in DoD settings. Using their expertise, knowledge, and access to key stakeholders, the MTFEC provided the PMC3 with up-to-date information on competing trials at specific MTF-recruitment sites. The MTFEC hosted regular monthly conference calls to facilitate exchange of key site-specific information among its members and provided this information to the PMC.

### Remediation phase

#### Addressing overlap with PMC trials

While the overarching goal of remediation was to assist PIs in meeting recruitment goals and avoiding contamination in pain interventions, secondary goals included fostering a shared sense of community, reducing competition among PCTs, and maintaining collegiality within the PMC. A PMC facilitator (a PI from the PMC3 with expertise in clinical-trial design) worked with PMC PIs to develop a plan to resolve issues surrounding site overlap, serving as an independent party to negotiate competing interests impartially and to maintain a collaborative spirit. In addition, the MTFEC moderated MTF-related issues. Together, the MTFEC and PMC3 operated as joint facilitators allowing the PMC PIs to identify, negotiate, and agree upon viable solutions. Given the involvement of 20 PMC PIs located across the country, the most efficient format for shared decision-making was small-group discussions hosted virtually. Prior to each discussion, the PMC3 provided the following information to PMC PIs: (i) summary of overlapping MTFs and VAMCs, (ii) inclusion/exclusion criteria and recruitment plans of proposed studies, (iii) summary of the ClinicalTrials.gov search, and (iv) a list of potential strategies to address conflict.

Because individual PIs were most knowledgeable about their own pain populations, as well as how and from where potential participants would be recruited into their studies, the facilitator moderated discussions—identifying sites with overlap, describing concerns, and presenting general remediation strategies without dictating any specific course of action. Discussions began with PIs sharing information about their recruitment methods and design, including details not readily apparent from each protocol, given that many were still being developed. This activity allowed the PIs to determine the likelihood that overlap concerns were real, and if so, how to work together with the facilitator to determine an appropriate remedy. For example, in one instance, PIs recruiting participants from the same site determined that they could use different clinics (primary care vs. specialty) with minimal impact on outcomes. Such information was known only by those PIs, each of whom had a shared interest in ensuring rigorous study design and recruitment practices.

#### Addressing overlap with non-PMC trials

Information about the 49 non-PMC pain trials obtained from ClinicalTrials.gov did not prove to be informative. Based on PIs’ experience during the site-selection process, the non-PMC pain trials identified by ClinicalTrials.gov neither provided new information (i.e., they were studies already familiar to a PI) nor captured all relevant site-level activities (e.g., pain initiatives in pre-planning stages, registration status, or quality-improvement design). Collaboration with the MTFEC, in contrast, provided a viable path to identify active pain trials and other pain interventional initiatives at each of the participating MTFs, through direct engagement with clinical leaders (directors of pain, musculoskeletal, and integrative medicine) at those sites [7]. The MTFEC communicated regularly with DoD PIs to discuss recruitment sites and current/anticipated obstacles and to develop troubleshooting plans.

## Results

The PMC has 61 recruitment sites—seven are MTFs, 51 are VAMCs, and three are civilian facilities. Our analysis revealed that 17 PMC sites (28%) overlapped. Four PCTs had five overlapping MTFs (Table 2), and eight PCTs had 12 overlapping VAMCs (Table 3). We developed three general strategies to avoid these overlaps: (i) modify exclusion criteria (e.g., exclude specific pain populations, participants enrolled in other pain trials, and participants receiving concomitant pain therapies), (ii) coordinate recruitment efforts (e.g., offer choice of enrollment, communicate and avoid enrollment with other PMC trials), and/or (iii) replace or avoid the overlapping site. In most cases, multiple strategies to avoid overlap were required within and across trials.

**Table 2:** Military Treatment Facility overlap in the Pain Management Collaboratory pragmatic trials

PMC trial number	Brooke Army, San Antonio, TX	Walter Reed, Bethesda, MD	Naval Medical, San Diego, CA	Darnall, Fort Hood, TX	Wilford Hall, San Antonio, TX	Population and setting	Target sample size and location
10	x	x	x			Surgical patients with acute pain (inpatient setting)	*N* = 592 4 MTF sites
11	x	x	x			PT care for LBP (outpatient setting)	*N* = 4672 3 MTF 2 VA sites
9	x	x		x	x	Chronic pain (PCP clinic)	*N* = 800 4 MTF sites
5	x			x	x	Acute and chronic LBP (PCP clinic)	*N* = 1200 4 MTF sites
Number of non-PMC pain studies	9	6	9	0	0		

x indicates overlapping site

*CA* California, *LBP* low back pain, *MD* Maryland, *N* number, *MTF* Military Treatment Facility, *PMC* Pain Management Collaboratory, *PT* physical therapy, *PCP* primary care provider, *TX* Texas, VA Veterans Affairs

**Table 3:** VA Medical Center overlap in the Pain Management Collaboratory pragmatic trials

PMC trial number	Tampa, FL	San Antonio, TX	Little Rock, AK	Portland, OR	St. Louis, MO	West Haven, CT	Los Angeles, CA	Minneapolis, MN	Durham, NC	Salisbury, NC	Boston, MA	Charleston, SC	Population and setting	Target sample size and location
11	x	x											PT care for LBP (outpatient setting)	*N* = 4672 3 MTF sites 2 VA sites
7	x	x	x	x	x					x	x		Chronic MSD (secondary data analysis)	*N* = 18,000 18 VA sites
6	x		x	x	x	x							Chronic pain (outpatient clinics)	*N* = 763 6 VA sites
2						x					x		Chronic MSD (PCP and mental health clinics)	*N* = 1100 8 VA sites
3						x	x	x					Chronic LBP (chiropractic clinics)	*N* = 766 4 VA sites
8							x	x	x				Chronic MSD (outpatient setting)	*N* = 750 4 VA sites
4									x	x		x	Acute and chronic LBP (outpatient clinics)	*N* = 1680 9 VA sites (16 clinics)
1												x	Chronic MSD (PCP and mental health clinics)	*N* = 764 9 VA sites
Number of non-PMC pain studies	0	2	0	1	0	9	0	4	1	0	7	1		

x indicates overlapping site

*AK* Arkansas, *CA* California, *CIH* complementary integrative health, *CT* Connecticut, *FL* Florida, *LA* Los Angeles, *LBP* low back pain, *MA* Massachusetts, *MD* Maryland, *MN* Minnesota, *MSD* musculoskeletal disorder, *MTFEC* Military Treatment Facility, *N* number, *NC* North Carolina, *PMC* Pain Management Collaboratory, *PT* physical therapy, *PCP* primary care provider, *SC* South Carolina, *TX* Texas, *VA* Veterans Affairs

All remediation strategies for MTFs and VAMCs are summarized in Tables 4 and 5, respectively. Our results show that modifying exclusion criteria alone was an effective strategy to eliminate overlap at 10 sites (Table 6). Other effective strategies include coordination of recruitment efforts alone (effective at three sites), site exclusion (effective at one site), and a combination of strategies (effective at two sites). Of note, modifying exclusion criteria also helped PIs address the potential for overlap with non-PMC pain trials, but was used judiciously because this strategy does not align with the philosophy behind pragmatic trial design. Coordination of recruitment efforts was the second most effective approach to averting research-site overlap. This coordination strategy, made possible through the PMC, was more frequently used by PIs enrolling participants at MTFs—an important consideration to ensure successful enrollment due to the limited availability of performance sites. Additionally, the Chair of the MTFEC encouraged the PIs to rely strongly on their inter-DoD networks. These internal connections were also helpful for enabling the MTFEC to avoid overlap with non-PMC trials.

**Table 4:** Remediation strategies for Military Treatment Facilities (MTFs) in the Pain Management Collaboratory (PMC)

MTF	Remediation plan	
Brooke Army, San Antonio, TX	PMC #5	• Coordinate site-recruitment efforts by communicating locations and avoiding clinics with PMC #9. • Coordinate site-recruitment efforts by communicating locations of clinics to PMC #11.
	PMC #9	• Coordinate site-recruitment efforts by communicating locations and avoiding clinics with PMC #5. • Coordinate site-recruitment efforts at site by communicating locations of clinics to PMC #11.
	PMC #10	• Exclude patients with chronic pain > 3 months of any severity or anatomic location.
	PMC #11	• Coordinate site-recruitment efforts by avoiding clinics being used by PMC #5. • Coordinate site-recruitment efforts by avoiding clinics being used by PMC #9.
Navy Medical, San Diego, CA	PMC #10	• Exclude patients with chronic pain > 3 months of any severity or anatomic location.
	PMC #11	• No action required.
Walter Reed, Bethesda, MD	PMC #9	• Avoid—do not recruit at this site.
	PMC #10	• Exclude patients with chronic pain > 3 months of any severity or anatomic location.
	PMC #11	• No action required.
Darnall, Fort Hood, TX	PMC #5	• Coordinate site-recruitment efforts by communicating locations and avoiding clinics with PMC #9.
Wilford Hall, San Antonio, TX	PMC #9	• Coordinate site-recruitment efforts by communicating locations and avoiding clinics with PMC #5.

**Table 5:** Remediation strategies for VA Medical Centers (VAMCs) in the Pain Management Collaboratory (PMC)

VAMC	Remediation plan	
Tampa, FL San Antonio, TX Little Rock, AK Portland, OR St. Louis, MO	PMC #6	• Exclude participants enrolled in other ongoing pain intervention trials.
	PMC #7	• No action required. Not anticipated to be an issue with other PMC trials.
	PMC #11	• No action required. Not anticipated to be an issue with other PMC trials.
VACT, CT	PMC #2	• Exclude participants enrolled in other ongoing pain intervention trials. • Exclude participants using ≥ 3 non-pharmacologic pain treatment modalities.
	PMC #3	• Exclude participants enrolled in other ongoing pain intervention trials
	PMC #6	• Exclude participants enrolled in other ongoing pain intervention trials
Greater LA, CA Minneapolis, MN	PMC #3	• Exclude participants enrolled in other ongoing pain intervention trials.
	PMC #8	• No action required.
Durham, NC	PMC #4	• No action required. Not anticipated to be an issue with other PMC trial.
	PMC #8	• No action required. Not anticipated to be an issue with other PMC trial.
Salisbury, NC	PMC #4	• Avoid—do not recruit at this site.
	PMC #7	• No action required.
Boston, MA	PMC #2	• Exclude participants enrolled in other ongoing pain intervention trials. • Exclude participants using ≥ 3 non-pharmacologic pain treatment modalities.
	PMC #7	• No action required.
Charleston, SC	PMC #1	• Coordinate site-recruitment efforts by avoiding clinics used by PMC #4.
	PMC #4	• Coordinate site-recruitment efforts by communicating locations with PMC #1.

*AK* Arkansas, *CA* California, *CT* Connecticut, *FL* Florida, *LA* Los Angeles, *MA* Massachusetts, *MD* Maryland, *MN* Minnesota, *NC* North Carolina, *PMC* Pain Management Collaboratory, *SC* South Carolina, *TX* Texas, *VA* Veterans Affairs, *VACT* VA Connecticut Healthcare System, *WV* West Virginia

**Table 6:** Overall summary of remediation strategies

Remediation strategy	Number of sites remedied
Modify exclusion criteria alone	10
Modify exclusion criteria plus coordinate recruitment efforts	1
Modify exclusion criteria plus avoid site	1
Coordinate recruitment efforts alone	3
Coordinate recruitment plus avoid site	0
Avoid—do not recruit at site alone	1
No action required	1

Another potential coordination strategy considered was to offer research participants a choice of PMC pain trials at their time of enrollment. This approach was deemed impractical based upon excessive regulatory and logistic hurdles, and thus, it was abandoned. We learned that asking PIs to select an alternative site was overly burdensome for most (and thus, only used by two PIs to avoid recruitment issues at one site). It is possible, however, that we did not capture all these instances, since some PIs used the information from the Excel pivot table to avoid selecting recruitment sites with competing PMC pain trials while still working through their site-selection process.

As noted earlier, our attempts to derive strategies to identify and mitigate overlap of PMC trials with non-PMC trials appeared to be largely unsuccessful and possibly unnecessary—in most cases, PIs were aware of such trials external to the PMC. However, a thorough scan of potentially overlapping non-PMC pain trials exceeded the resources available within the PMC. Going forward, four of the 11 PCT PIs anticipated that they would collect information on non-PMC pain trials to use as a covariate in a sensitivity analysis to identify potential for contamination from site overlap. Previous reports have discussed using a factorial design approach to glean further statistical insights into addressing this problem [8].

## Discussion

By design, PCTs recruit a diverse study population and usually have broad inclusion criteria, trading internal validity for greater generalizability, toward prompt implementation of effective interventions in clinical settings that mimic those of the trials themselves [9]. There is great urgency for delivering evidence-based nonpharmacological approaches and integrated pain-care models to military service members and veterans—two groups especially vulnerable due to a high prevalence and complexity of pain and pain management [10]. The PMC’s focus on PCTs to evaluate nonpharmacological approaches conducted in DoD and VA health care systems is intended to inform decision-makers (veterans and military service members and their dependents, providers, administrators, and policy makers) about the relative benefit, risks, and feasibility of these approaches when delivered in these health care systems. The main strength of the PMC is its shared focus among 11 large-scale, multi-site PCTs; however, that feature is also a weakness due to shared timelines and similar study populations targeted by different PCT PIs. In recognition of the potential for participant competition and study contamination from overlapping recruitment sites, the PMC developed a plan to address this priority issue soon after the program’s launch, to begin the PMC’s 2-year planning phase, and follow-up is planned after 2 years. As we have demonstrated, our efforts have been successful; the PMC3 identified and corrected potential competition or contamination at 16 of 17 (94%) PMC trial locations.

We believe that beyond specific strategies, the organizational structure and existing relationships within the PMC contributed to our success. The time and energy used to create the PMC allowed the PCT PIs to establish familiarity, build trust, and develop a shared sense of community. Other key determinants for successfully averting research-site overlap were gathering the right type of information for PIs to consider and discuss, as well as offering an impartial moderator with expertise in clinical-trial design to resolve potential conflicts. The PMC3 itself acted as a fair and trusted third party to help PIs negotiate a set of remediation strategies balanced across all PMC trials. The final strategy for addressing research-site overlap was endorsed by the primary PMC stakeholders: the PMC Steering Committee composed of the sponsors, trial PIs, and the PMC3.

The PMC3 learned several lessons from this exercise. These include the importance of prompt action; accurate and clear synthesis of information; and effective engagement of PIs, sponsors, and stakeholders. Above all, we learned the necessity of thinking ahead about potential pitfalls that could discount the validity of many person-years of work—and more pressingly, potentially delay delivery of effective treatments for people living with debilitating pain. The utility of PCTs is their ability to provide flexibility to adapt dynamically to results obtained in real-world conditions—thus requiring large and diverse participant populations to reflect what happens in real life. However, as for explanatory trials, the design and conduct of PCTs must adhere to the principles of scientific rigor to avoid participants either being double-counted or receiving more than one intervention, both of which would cloud data interpretation. The sponsors and PMC3 were quick to recognize the need for a well-articulated, collaborative process of shared decision-making to ensure that PIs are able to conduct their own research as well as preserve the integrity of the consortium’s shared vision.

Some activities in our information-gathering stage could have been streamlined or eliminated entirely. Gathering written recruitment plans about the PMC trials placed a burden on PIs and may have been unnecessary considering these details naturally arose in discussions on our many conference calls to develop a strategy to avert research-site overlap. Likewise, the ClinicalTrials.gov search was resource-intensive, involving several people in the PMC3 to identify parameters, pull/clean data, and synthesize information in a timely manner. Ultimately, none of the overlapping sites identified through the ClinicalTrials.gov search provided meaningful information as this information was either already known to PIs or not relevant. In retrospect, however, such search information could have been helpful if it was introduced further upstream in the pre-planning stage (before beginning the site-selection process).

Engaging PMC PIs, sponsors, and key stakeholders was critical for success, as each plays a vital but different role in the overall PMC effort. Individual PIs are the most knowledgeable about recruitment for their own PCTs, so they must be involved and invested in details of remediating site overlap. As important are people with comprehensive environmental knowledge and policy awareness, such as the MTFEC, who knew about programs and proposed initiatives that would affect recruitment more broadly for all PMC sites and who helped resolve MTF-specific issues. Input from sponsors and key stakeholders was also invaluable. For example, VA personnel identified the *Whole Health Evaluation Program* that would affect recruitment at six VAMCs leading to the PMC decision to delay recruitment at these sites until May 2020 when the evaluation was expected to be complete. Because the PMC only conducts pain-relevant PCTs in military and VA health care systems, the strategies we have identified and presented herein may not be suitable to other collaboratories that conduct PCTs in disparate disease areas and conditions. Finally, we note that the process we used and have detailed herein may have been difficult to pursue for explanatory trials with less flexibility in study design and participant recruitment.

## Conclusion

In reality, there is a continuum between pragmatic and explanatory approaches, and many individual trials are likely to possess some elements of both as appropriate to the research question at hand, the trial population, and other environmental issues [11]. Our approach to ensure scientific rigor while maximizing external validity among PMC PCTs demonstrates that relatively concrete steps, if articulated clearly and transparently, can help accomplish this goal. As with many elements of biomedical investigation, collaboration and mutual respect have been main drivers of our ability to optimize conditions for obtaining knowledge that can be put to use in the real world. Although we recognize that uptake of evidence-based knowledge is variable for a range of reasons beyond trial design and recruitment efforts, our hope is that lessons learned from the large and complex PMC effort will help other similar consortia navigate and avoid issues that may compromise data interpretation.

## Data Availability

Not applicable

## Abbreviations

PMC: The Pain Management Collaboratory
PCTs: Pragmatic clinical trials
DoD: US Department of Defense
VA: Department of Veterans Affairs
NIH: National Institutes of Health
MTFEC: Military Treatment Facility Engagement Committee
MTFs: Military Treatment Facilities
VAMCs: VA Medical Centers
PIs: Principal investigators
HHS: US Department of Health and Human Services
CMS: Centers for Medicare and Medicaid Services
NCT: National Clinical Trial number

## References

[1] Schwartz D, Lellouch J (1967). Explanatory and pragmatic attitudes in therapeutical trials. J Chronic Dis.

[2] Ford I, Norrie J (2016). Pragmatic trials. N Engl J Med.

[3] Tunis SR, Stryer DB, Clancy CM (2003). Practical clinical trials: increasing the value of clinical research for decision making in clinical and health policy. JAMA.

[4] Kerns R, Brandt C, Peduzzi P, NIH-DoD-VA Pain Management Collaboratory (2019). NIH-DoD-VA Pain Management Collaboratory. Pain Med.

[5] Pragmatic and Implementation Studies for the Management of Pain to Reduce Opioid Prescribing (PRISM): https://heal.nih.gov/research/clinical-research/prism. Accessed 30 Mar 2020.

[6] Krejci L, Carter K, Gaudet T (2014). The vision and implementation of personalized, proactive, patient-driven health care for veterans. Med Care.

[7] Coulter I, Hilton L, Walter J, Brown K (2016). Integrative pain management centers in the military: the challenges. Mil Med.

[8] Larntz K, Neaton JD, Wentworth DN, Yurik T (1996). Data analysis issues for protocols with overlapping enrollment. Stat Med.

[9] Ware JH, Hamel MB (2011). Pragmatic trials–guides to better patient care?. N Engl J Med.

[10] Institute of Medicine (2011). Relieving pain in America: a blueprint for transforming prevention, care, education, and research.

[11] Loudon K, Treweek S, Sullivan F, Donnan P, Thorpe KE, Zwarenstein M (2015). The PRECIS-2 tool: designing trials that are fit for purpose. BMJ.

